# A Network Pharmacology-Based Study on Vital Pharmacological Pathways and Targets of Eucommiae Cortex Acting on Osteoporosis

**DOI:** 10.1155/2022/8510842

**Published:** 2022-03-31

**Authors:** Libo Zhou, Tao Wu

**Affiliations:** ^1^Joint Surgery, Qinghai University, Qinghai 810001, China; ^2^Joint Surgery, Affiliated Hospital of Qinghai University, Qinghai 810001, China

## Abstract

**Background:**

Eucommiae Cortex is a Chinese herbal medicine with bone protective effects and treats osteoporosis. This study aimed to explore the pharmacological mechanisms of this complex mixture.

**Methods:**

The active compounds and disease targets involved in the study were obtained from publicly available websites and databases. Core target genes were identified by protein-protein interaction (PPI) network and topology analysis and mapped to critical components by a “component-target” regulatory network. Kyoto Encyclopedia of Genes and Genomes (KEGG) Pathway analysis and Gene Ontology (GO)analysis were performed to analyze the biological processes of target genes. Moreover, we carried out molecular docking, cell experiments, and quantitative real-time PCR to propel the research forward.

**Results:**

Eucommiae Cortex contained 28 active ingredients and 85 potential targets for antiosteoporosis. PPI networks and topology analysis screened 17 core target genes. The therapeutic mechanism involves a series of biological reactions, including host-virus response, chemical stress, oxidative stress, and cell cycle control. The KEGG enrichment illustrated that the MAPK signaling pathway might play a significant role. The final experiment detected ten genes (EGF, AKT1, JUN, MAPK8, MAPK1, CASP3, FOS, VEGFA, EGFR, and MYC) and three compounds (quercetin, kaempferol, and beta- carotene) in the MAPK pathway. Firstly, the CCK-8 and ALP activity test results showed that three compounds could enhance the proliferation and differentiation of osteoblast-like MC3T3-E1 cells. Secondly, molecular docking confirmed the favorable binding potential. Subsequently, we observed that adding 1∗10^−6^ mol/L quercetin, 1∗10^−5^  mol/L kaempferol, and 1∗10^−5^  mol/L beta-carotene activated the ERK/JNK cascades and the heterodimer complex AP-1(Fos/Jun) in the MAPK pathway.

**Conclusion:**

MAPK pathway might provide an essential mechanism for the antiosteoporosis effect of Eucommiae Cortex. Eucommiae Cortex and its active ingredients have the potential to treat osteoporosis.

## 1. Introduction

Osteoporosis is a common chronic metabolic bone disease with considerable social and health implications. The prevalence of osteoporosis increased with age, bringing safety hazards to the elderly, including signs of augmented fracture [1]. According to the latest statistics of the International Osteoporosis Foundation, one-third of women over 50 years old and one-fifth of men will encounter osteoporosis fractures in their life [2]. Osteoporotic fractures account for 0.83% of the global burden of noncommunicable diseases, and this share is likely to rise [3]. Unfortunately, osteoporosis requires lifelong medication to prevent fractures. A recent meta-analysis found that bisphosphonate benefited 1% of potential female patients after a minimum of 12.4 months of continuous use [4]. Although the advent of newer drugs such as teriparatide and denosumab ushered at the dawn of antiosteoporosis treatment, evidence of long-term benefit is absent [5]. The long-term efficacy and safety of first-line therapy for osteoporosis remain an issue of concern. There is a strong demand to develop drugs with more moderate side effects and boost bone metabolism over the long term.

Eucommiae Cortex, a traditional Chinese medicine (TCM), is widely used to treat osteoporosis, lumbago, and hypertension in TCM clinics. In the *Compendium of Materia Medica*, it is recorded that *Eucommia* can nourish the liver and kidney and treat waist and knee pain. Eucommiae Cortex 60% ethanol extract can remarkably inhibit the osteopenia caused by tail suspension, protect the microstructure of femoral trabecular bone, and improve the biomechanical properties of rat femur [6]. In particular, Eucommiae cortex could induce osteogenic differentiation of bone marrow mesenchymal stem cells through Wnt/*β*-catenin, MAPK, and Rho A/ROCK signaling pathways [7]. Eucommiae Cortex has a variety of active components and complex mechanisms of action. It is indispensable for screening and validation before Eucommiae Cortex is considered a latent medication. Network pharmacology can establish systematic networks of drug components, targets, and diseases at different levels for screening. The research perspective of systems biology and the network pharmacology methods are more aligned with the characteristics of natural herbal medicine.

Based on network pharmacology and cell experiments, this study explored the important mechanism and targets of Eucommiae Cortex against osteoporosis. After obtaining the targets of Eucommiae Cortex and osteoporosis in authoritative public databases, we constructed a “component-target” regulatory network. The primary pathways are analyzed with topology analysis and biological function analysis. Subsequently, in silico and in vitro experiments, we verified that relative components and core target genes of the primary pathway play significant roles in the proliferation and differentiation of osteoblasts.

## 2. Materials and Methods

### 2.1. Acquisition of Active Drug Ingredients and Their Targets

TCMSP platform is a platform for drug screening and evaluation of herbal medicine systems. It provides information on essential characteristics of ADME, such as half-life, oral bioavailability (OB), drug-like properties (DL), and blood-brain barrier. In previous studies, OB and DL were two crucial criteria for drug screening [8]. Through the TCMSP platform, “du Zhong” and “Eucommiae Cortex”were used as the keywords for retrieval. The limiting conditions were set as OB > 30% and DL > 0.18, and obtained the information of all the chemical components of Eucommiae Cortex. The active ingredient targets were obtained from the Drugbank database and published literature. In addition, we searched the protein database ID from the Uniprot database.

### 2.2. Acquisition of Potential Therapeutic Targets of Eucommiae Cortex for Osteoporosis

The keyword “Osteoporosis” was sought in the GeneCards database and online human Mendelian inheritance (OMIM) database. Further, obtained common target genes of drugs and diseases serve as potential therapeutic targets for antiosteoporosis.

### 2.3. Construction of a “Component-Target” Regulatory Network

The active ingredients and potential antiosteoporosis targets were imported into Cytoscape 3.7.2 software to build a “component-target” regulatory network. This network can guide each compound and target to find the corresponding match.

### 2.4. Protein-Protein Interaction (PPI) Network and Topology Analysis

Potential therapeutic targets were retrieved from the STRING database and obtained the interaction relationships. The protein interaction network was drawn by Cytoscape software. The TSV files exported in the STRING database are imported into the Cytonca plugin. A total of six degrees are available for comprehensive screening of target genes, including betweenness (BC), clotonca, degree (DC), eigenvector (EC), local average connectivity- (LAC-) based method, and network (NC) [9]. The six-degree values represent the influence of nodes at different levels and choose the median value of each degree as the filtering condition of topology analysis.

### 2.5. Analysis of Biological Processes and Pathways of Core Targets

R software was used to study the core targets of Kyoto Encyclopedia of Genes and Genomes (KEGG) Pathway analysis and Gene Ontology (GO) analysis, with a *P* value <0.05 as the screening condition. Bar chart and bubble chart show the top 20 results.

### 2.6. Molecular Docking

The compounds' structures were obtained from PubChem's bioassay database. The compounds were executed to energy minimization using the software Chem3D (version 17.0) and saved in the PDB format. We set rotatable keys of the ligands and saved them as a PDBQT file for semiflexible docking using the software AutoDockTools-1.5.6. We downloaded the structures in the PDB format of the targets from the Uniport database. The receptor proteins were processed by Pymol software to delete the ligands bound initially to the receptors. We selected the docking region of the proteins, including the central site (*X*, *Y*, and *Z*) and the docking size on the *X*, *Y*, and *Z* axis. To display the docking results, open the receptor PDB file and the ligand PDBQT file by Pymol software simultaneously. AutoDock Vina was used to conduct molecular docking.

### 2.7. Cell Experiment

#### 2.7.1. Materials and Samples

**Figure 1:**
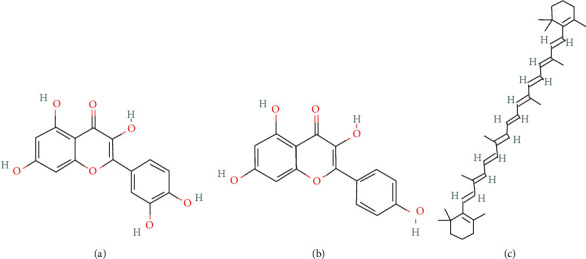
Chemical formula of (a) quercetin, (b) kaempferol, and (c) beta-carotene.

Cell Counting Kit-8 (CCK-8) was purchased from Beijing Solar-bio Science & Technology Co., Ltd. (Beijing, China). ALP kit was bought from Jiancheng Bioengineering Institute (Nanjing, China). MC3T3-E1 cells were purchased from the Cell Culture Collection Committee of the Chinese Academy of Sciences (Shanghai, China). DMEM and FBS were purchased from Life-iLab (Shanghai, China). TRIzol reagent was ordered from Invitrogen (Carlsbad, CA, USA). SYBR Green Pro Tap was brought from AG (Shanghai, China). Primer was synthesized by Sangong Bioengineering (Shanghai, China). Quercetin, kaempferol, and beta-carotene were ordered from Sigma-Aldrich, Merck KGaA (Darmstadt, Germany). The molecular formula is shown in Figure 1.

#### 2.7.2. Cell Culture

MC3T3-E1 cells, established from C57BL/6 mouse calvaria, were outsourced from the Cell Culture Collection Committee of the Chinese Academy of Sciences (Shanghai, China). These cells were cultured in a DMEM medium (Gibco, Grand Island, NY, USA), supplemented with 10% fetal bovine serum (FBS, Gibco, Grand Island, NY, USA) at 37°C in a chamber with 5% CO2.

#### 2.7.3. CCK-8 and ALP Activity Assay

As for CCK-8, MC3T3-E1 cells were cultured for 24 h in 96-well plates at a density of 5 × 10^3^ cells/well, and then treated with quercetin at concentrations of 10^−9^, 10^−8^, 10^−7^, 10^−6^, 10^−5^, and 0 mol/L for 24, 48, and72 h in DMEM. Then, 100 *μ*L fresh medium containing 10 *μ*L CCK-8 solution was added to each well, and the cells were incubated at 37°C for one h. The study was using a microplate reader to measure the absorbance at 450 nm.

As for the ALP activity assay, MC3T3-E1 cells were cultured for 24 h in 24-well plates at a density of 2 × 10^4^ cells/well and then treated with quercetin at concentrations of 10^−9^, 10^−8^, 10^−7^, 10^−6^, 10^−5^, and 0 mol/L for 24, 72, and 120 h in DMEM. Each hole cell was gently washed thrice with phosphate-buffered saline and then lysed with 500 *μ*L 0.1% Triton X-100. The ALP activity assay kit was used to determine ALP activity in the lysate. The micromoles of p-nitrophenol liberated per-nanogram protein expressed the ALP activity. Kaempferol and beta-carotene adopt the same strategy.

#### 2.7.4. Total RNA Isolation and Quantitative Real-Time PCR Analysis

Total RNA was isolated from osteoblast cell cultures using TRIzol reagent. Then, RNA samples were reverse-transcribed into cDNA. cDNA is used as the template for qRT-PCR analysis of target genes using SYBR Green Pro Tap (AG, China). Specific primers are listed in Table 1. The following parameters for PCR amplification were used: 94°C 30 sec, 30 cycles of 98°C for 10 sec, 55°C for 30 sec, and 72°C for 60 sec, and a final extension step of 72°C 120 sec. Relative mRNA expression levels were calculated by the 2-*ΔΔ*Ct method. Each sample was assayed at least five times.

### 2.8. Statistical Analysis

Statistical analysis was processed with Prism 8 software and R4.1.0software. Data were expressed as the mean ± SD and analyzed using Student *t*-test or one-way ANOVA. Differences between groups were considered statistically significant if values of *P* < 0.05.

## 3. Results

### 3.1. Screening of Active Ingredients and Target Genes

In the TCMSP database, Eucommiae Cortex contains 28 active ingredients in total, with OB ≥ 30% and DL ≥ 0.18 as the conditions (Table 2). Then, the DrugBank database and Uniprot database predicted the targets. Eventually, a total of 181 drug targets were obtained.

### 3.2. Eucommiae Cortex Potential Therapeutic Targets

**Figure 2:**
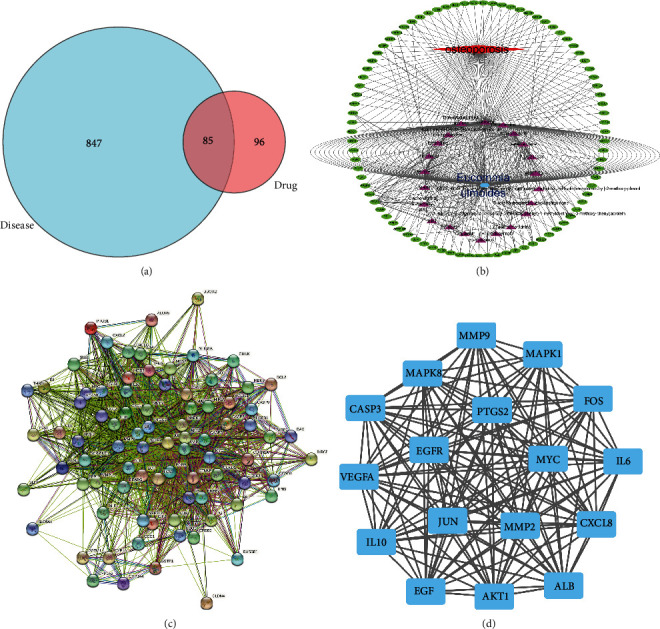
(a) Venn diagram. It displays the interset of compounds targets related to osteoporosis targets. (b) “Component-target” regulatory network. The regulatory network shows the matched relationship between the active components and the osteoporosis genes. The blue symbol in the inner ring represents herbal Eucommiae cortex. The rose symbols in the outer circle represent 28 active ingredients. The red symbol represents osteoporosis, and the green signs in the outer ring represent 85 intersection genes. (c).PPI network. (d). Topological analysis.

A total of 932 targets of osteoporosis were obtained in the Genecards database and OMIM database. After comparison with drug targets, 85 intersection targets were derived (Table 3, Figure 2(a)).

### 3.3. A “Component-Target” Regulatory Network

The “component-target” regulatory network represents the correspondence between active compounds and antiosteoporosis targets (Figure 2(b)). Among them, quercetin and kaempferol have the most related marks.

### 3.4. PPI Network and Topological Analysis

PPI network showed the interaction between potential therapeutic targets, with 84 nodes (one of the targets is hidden because of no protein interaction) and 1516 edges (Figure 2(c)). A subsequent topology analysis filtered 84 nodes. The median value of BC, CC, DC, LAC, and NC are taken for filtering (BC: 21.594879345; CC: 0.6264240145; DC: 34; EC: 0.0984259805; LAC: 26.26559715; NC: 28.311158725). 36 nodes and 563 edges were obtained. Then, take the median filter again (BC: 3.2453257765; CC: 0.897435897; DC: 31; EC: 0.1647364195; LAC: 27.51612903; NC: 29.57936678); 17 nodes and 136 edges were eventually identified (Figure 2(d)). According to the “component-target” regulatory network, quercetin, kaempferol, and beta-carotene were most associated with 17 core targets, which were selected for subsequent verification.

### 3.5. GO and KEGG Analysis

**Figure 3:**
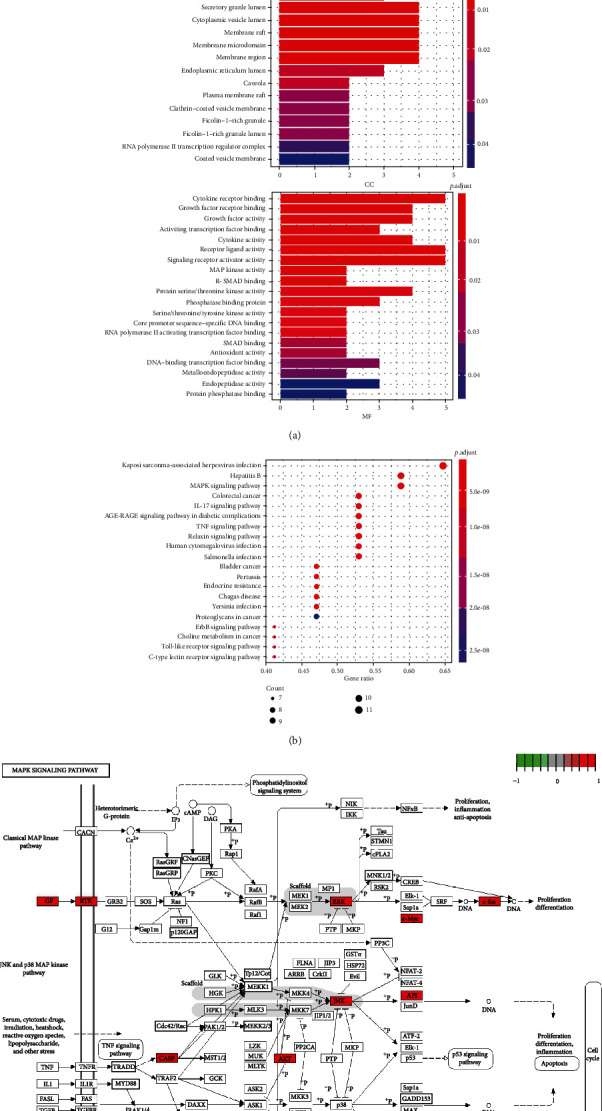
(a) The BP, CC, and MF column color symbolizes the enrichment significance based on the corrected *P* value. The horizontal axis represents the number of genes enriched on each item. (b) The vertical axis of the KEGG bubble diagram represents the enrichment degree according to the corrected *P* value. Moreover, the horizontal axis shows the gene proportion enriched in each entry. (c) MAPK signaling pathway. The relevant targets of the MAPK signaling pathway are marked red.

GO analysis illustrates gene function on three levels: biological process (BP), cellular component (CC), and molecular function (MF) (Figure 3(a)). BP mainly involves cellular response to chemical stress, oxidative stress, and metal ion. CC is mainly related to the vesicle lumen, platelet alpha granule lumen, and secretory granule lumen. MF is involved primarily in cytokine receptor, growth factor receptor, activating transcription factor binding, MAP kinase activity, and R−SMAD binding. According to KEGG enrichment results, the mechanisms of Eucommiae Cortex of antiosteoporosis are mainly concentrated in Kaposi sarcoma-associated herpesvirus infection, MAPK signaling pathway, and hepatitis B signaling pathway (Figure 3(b)). Combined with GO, KEGG results, and disease-related biological knowledge, the MAPK signaling pathway was most valuable in developing osteoporosis. Ten genes from the MAPK signaling pathway were selected (Figure 3(c)).

### 3.6. Molecular Docking

**Figure 4:**
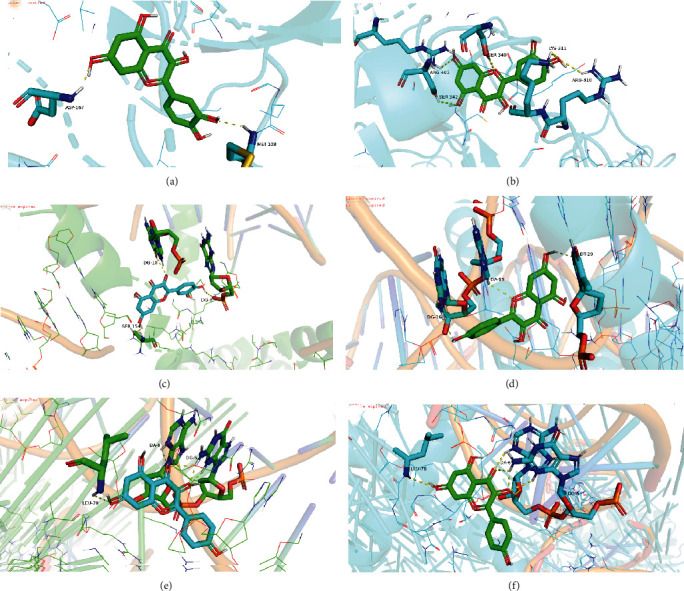
Molecular docking: (a) quercetin-ERK2, (b) quercetin-EGF, (c) quercetin-C-FOS, (d) kaempferol-AKT, (e) kaempferol-CASP3, and (f) kaempferol-MAPK8.

Molecular docking mimics the interaction of small ligands with large receptor protein molecules. The affinity can be predicted by calculating the binding energy. Smaller binding energy indicates a more stable structure. Quercetin, kaempferol, and beta-carotene have good binding potential with MAPK pathway target genes (Table 4, Figure 4).

### 3.7. The Proliferation of MC3T3-E1 Cells

**Figure 5:**
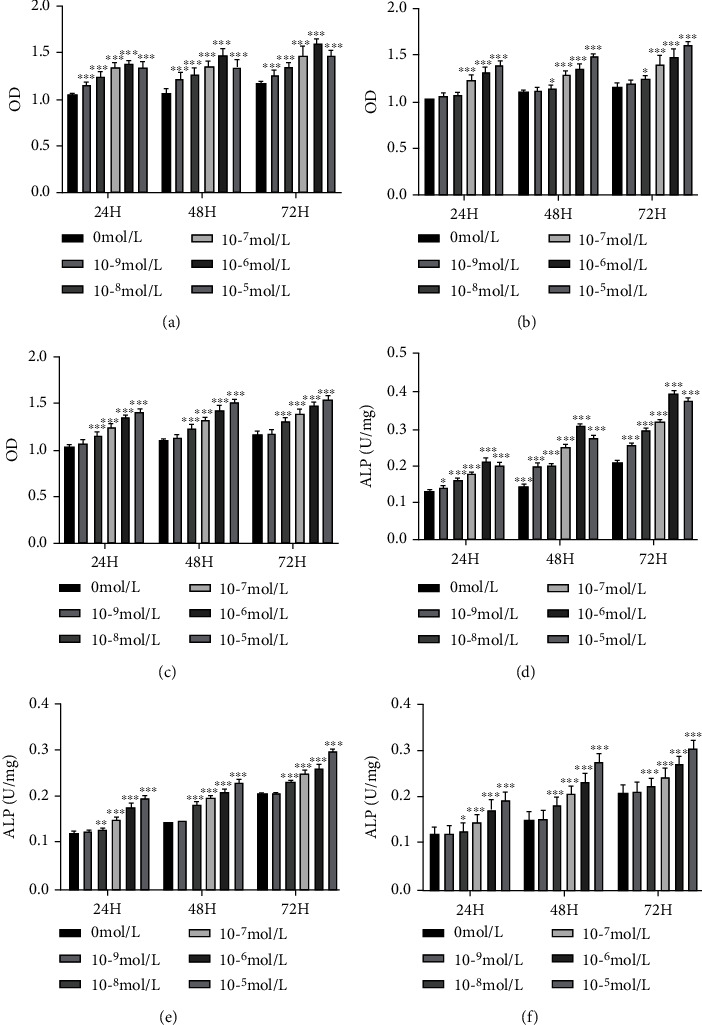
(a)–(c). CCK-8 detect cell viability: (a) quercetin, (b) kaempferol, and (c) beta-carotene. (d)–(f) ALP activity detect cell differentiation: (d) quercetin, (e) kaempferol, and (f) beta-carotene. *n* = 5; Student *t*-test vs. 0 mol/L; ∗*P* < 0.05, ∗∗*P* < 0.01, ∗∗∗*P* < 0.001.

As shown in Figures 5(a)–5(c), quercetin, kaempferol, and beta-carotene could vastly boost the proliferation in MC3T3-E1 cells. Moreover, it is proportional to time. When the concentration of quercetin, kaempferol, and beta-carotene was 1∗10^−6^ mol/L, 1∗10^−5^ mol/L, and 1∗10^−5^ mol/L, respectively, the maximum proliferation capacity was obtained.

### 3.8. The ALP Activity of MC3T3-E1 Cells

ALP is a glycoprotein secreted by osteoblasts, indirectly reflecting differentiation and maturity. As shown in Figures 5(d)–5(f), at 24, 48, and 72 h, the activity of ALP increased with time. As for quercetin, the concentration of 1∗10^−6^ reached the ALP activity peak, and differentiation was the highest. At 10^−5^, it was flat or slightly decreased. The optimal concentration of 1∗10^−6^ was selected. As for kaempferol and beta-carotene, the concentration gradient of 1∗10^−5^ is the optimum selection.

### 3.9. qPCR Validation of the Core Genes

**Figure 6:**
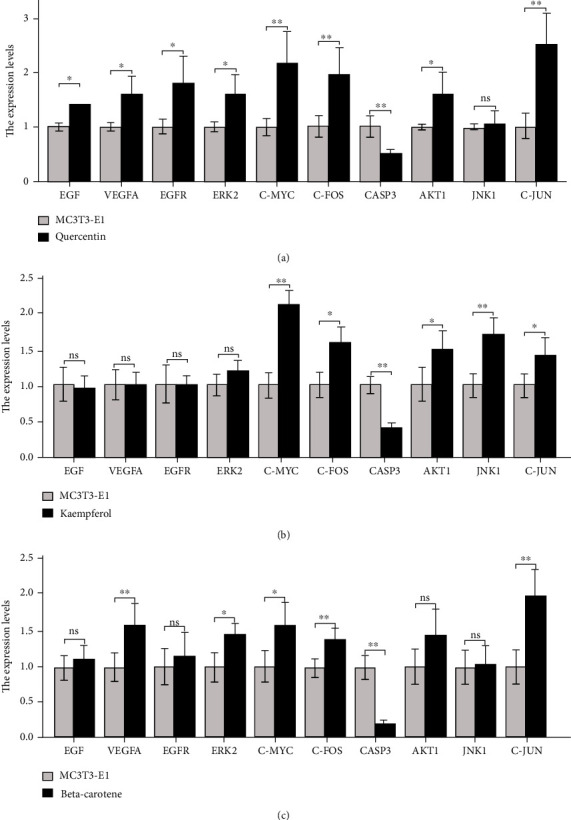
The effects of quercetin, kaempferol, and beta-carotene treatment on the mRNA expression of osteoblast: (a) quercetin, (b) kaempferol, and (c) beta-carotene. *n* = 5 vs. MC3T3-E1; ns: no significant; ∗*P* < 0.05, ∗∗*P* < 0.01, ∗∗∗*P* < 0.001.

The CCK-8 and ALP activity test results showed quercetin at 1∗10^−6^ mol/L, kaempferol at 1∗10^−5^ mol/L, and beta-carotene at 1∗10^−5^ mol/L be selected to verify. As shown in Figure 6, the addition of the three compounds amplified the transcription factor complex (Jun/FOS), thus playing a role in promoting proliferation and differentiation. Quercetin and beta-carotene mainly passed through the MAPK1/ERK2 pathway, while kaempferol passed through the MAPK8/JNK1 pathway.

## 4. Discussion

After entering old age, the body will slow down the production of new bone tissue. The etiology and pathogenesis of osteoporosis are frustratingly complex. Some of the leading causes may be the low level of sex hormones, the lack of differentiation of bone marrow stromal cells into osteoblasts, and the breaking of the dynamic balance between osteoclast bone resorption and osteoblast bone remodeling [10]. A recent review highlighted type I collagen processing, Wnt signaling, TGF-*β* signal transduction, RANKL rank system, and mechanical injury of bone cells [11]. Our study found that the MAPK pathway has substantial value in treating osteoporosis, particularly related to the pharmacodynamic effect of Eucommiae Cortex.

Network pharmacology combines molecular biology, genetics, and computer science to collect, analyze, mine, and deploy biological information. Behind diseases and natural drugs backgrounds, this technology can better predict and classify various therapeutical targets and interactions [8]. In previous studies, Eucommiae Cortex presented a significant bone protective effect on rats induced by lead acetate, promoting bone formation and inhibiting bone resorption [12]. However, the specific mechanisms and targets of action remain unclear. We explore 85 potential therapeutical targets through the “component-target” regulatory network, including apoptosis-related protein, growth factor, inflammatory factors, and protein kinase. Further, PPI analysis and topology analysis screened 17 core targets. According to the enrichment of biological functions and the supplement of existing literature, core targets are widely involved in the host-virus response, chemical stress, oxidative stress, and cell cycle control. KEGG analysis also demonstrated that the antiosteoporosis effect of Eucommiae Cortex was connected to virus infection and cell signal transduction, among which the MAPK pathway was the most valuable. There are three active compounds that correlated with the MAPK pathway—quercetin, kaempferol, and beta-carotene.

Mitogen-activated protein kinase (MAPK) cascades are essential signaling pathways that regulate various cellular processes, including proliferation, differentiation, and apoptosis [13]. According to our analysis results, Eucommiae Cortex is mainly related to the two branches, extracellular signal-related kinase (ERK) and Jun amino-terminal kinase (JNK). ERK signal is an indispensable member of cell development. It responds to various stimuli such as aging, trauma, and infection and drives biochemical activities for organ regeneration [14]. Growth factors can activate the ERK2/MAPK1 to induce cell proliferation and differentiation [15]. Our qPCR experiments reflected that quercetin simultaneously activated pro-epidermal growth factor (EGF), vascular endothelial growth factor A (VEGFA), and ERK2 genes. While beta-carotene only activated VEGFA and ERK2, JNK activity is crucial in the late differentiation of osteoblasts [16]. According to transcriptomic analysis, JNK1/MAPK8 was desired to up-regulate several osteoblast-derived angiogenic factors and was the critical mediator of osteoblast function [17]. Only kaempferol-stimulated osteoblasts showed JNK1 up-regulation.

Activator protein 1 (AP-1) transcription factor is a dimer complex composed of Jun and Fos proteins [18]. It is the downstream executive element of the MAPK pathway. AP-1 acts as a valid regulator in osteoblast differentiation and proliferation; under physiological conditions, the activity of AP-1 is determined by parathyroid hormone and transforming growth factor-*β* and 1,25-dihydroxy vitamin D [19]. Rainer Zenz [20] reviewed the capacity of AP-1 in bone immunological therapy. The therapeutic interventions on AP-1 signal transduction might provide potent weapons for treating low bone mass diseases. After three compounds stimulated osteoblasts, c-jun and c-fos were amplified in our study. Binding properties in vitro also demonstrated that the three compounds have superior binding force with Jun and FOS. This research provides indirect evidence for the study of targeting AP-1 transcription factors.

Quercetin and kaempferol are natural flavonoids found in a variety of phytomedicine. They participate in bone remodeling through the methods of inhibiting adipogenesis, inflammation, oxidative stress, and osteoblast apoptosis [21]. Beta-carotene is likewise salutary for maintaining bone health. Recent clinical studies have shown that higher beta-carotene intake was associated with a lower risk of osteoporosis and fractures [22]. Beta-carotene treatment also increased the expression of osteopontin and ALP, markers of bone differentiation [23]. Although the values of the three compounds as osteoprotective agents have received some attention, their effects on osteoblast proliferation and differentiation have not been carefully evaluated. Our research has proved that quercetin, kaempferol, and carotene can promote the proliferation and differentiation of osteoblasts. After being stimulated by the three compounds, the proliferation rate and ALP activity of MC3T3-E1 cells increased gradually at 24, 48, and 72 hours. It is worth mentioning that all three compounds reduced the expression of apoptosis factor CASP3 in MC3T3-E1 cells. Therefore, we speculate that the three compounds can inhibit osteoblast apoptosis.

In summary, we found 17 core targets, and the MAPK signaling pathway contributed to Eucommiae Cortex in treating osteoporosis. Subsequently, cell experiments and qPCR detections confirmed that active components (quercetin, kaempferol, and beta-carotene) promote the proliferation and differentiation of MC3T3-E1 cells. Molecular docking simulates the binding state at the computer level. Based on the above evidence, we speculate that Eucommiae Cortex can be used as a potential drug to treat osteoporosis. Our research experiment still has many shortcomings, but it can provide a basis for developing therapeutic drugs for low bone mass diseases and bone repair.

## Figures and Tables

**Table 1:** Quantitative real-time PCR primer sequences.

Gene	FOR	REV
EGF	5′-TTGGTATGCAAGGATGTGTC-3′	5′-CCACTTTGCGAAGTAACTTGGTA-3′
EGFR	5′-GCCATCTGGGCCAAAGATACC-3′	5′-GTCTTCGCATGAATAGGCCAAT-3′
VEGFA	5′-CCCGGGCCTCGGTTCCAG-3′	5′-GTCGTGGGTGCAGCCTGGG-3′
MAPK1/ERK2	5′-GCTGAATCACATCCTGGGTA-3′	5′-CTGTTCCACGGGCACCTTATT-3′
MAPK8/JNK1	5′-GTGGAATCAAGCACCTTCACT-3′	5′-TCCTCGCCAGTCCAAAATCAA-3′
CASP3	5′-GAAATTGTGGAATTGATGCGTGA-3′	5′-CTACAACGATCCCCTCTGAAAAA-3′
AKT1	5′-GGACTACTTGCACTCCGAGAA-3′	5′-CATAGTGGCACCGTCCTTGATC-3′
c-JUN	5′-ACTCG-GACCTCCTCACCTCG-3′	5′-TGTTTAAGCTGTGCCACCTGTT-3′
FOS	5′-GCGAGCAACTGAGAAGAC-3′	5′-TTGAAACCCGAGAACATC-3′
c-MYC	5′-AACTTACAATCTGCGAGCCA-3′	5′-AGCAGCTCGAATTTCTTCCAGATAT-3′
*β*-Actin	5′-GTACTCTGTGTGGATCGGTGG-3′	5′-AACGCAGCTCAGTAACAGTCC-3′

**Table 2:** The available compounds of Eucommiae Cortex.

MOl ID	Molecule name	OB (%)	DL
MOL002058	40957-99-1	57.2	0.62
MOL000211	Mairin	55.38	0.78
MOL000358	Beta-sitosterol	36.91	0.75
MOL000422	Kaempferol	41.88	0.24
MOL004367	Olivil	62.23	0.41
MOL000443	Erythraline	49.18	0.55
MOL005922	Acanthoside B	43.35	0.77
MOL006709	AIDS214634	92.43	0.55
MOL007059	3-Beta-hydroxymethyllenetanshiquinone	32.16	0.41
MOL000073	Ent-epicatechin	48.96	0.24
MOL007563	Yangambin	57.53	0.81
MOL009007	Eucommin A	30.51	0.85
MOL009009	(+)-Medioresinol	87.19	0.62
MOL009015	(-)-Tabernemontanine	58.67	0.61
MOL009027	Cyclopamine	55.42	0.82
MOL009029	Dehydrodiconiferyl alcohol 4,gamma'-di-O-beta-D-glucopyanoside_qt	51.44	0.4
MOL009030	Dehydrodieugenol	30.1	0.24
MOL009031	Cinchonan-9-al, 6'-methoxy-, (9R)-	68.22	0.4
MOL009038	GBGB	45.58	0.83
MOL009042	Helenalin	77.01	0.19
MOL009047	(+)-Eudesmin	33.29	0.62
MOL009053	4-[(2S,3R)-5-[(E)-3-Hydroxyprop-1-enyl]-7-methoxy-3-methylol-2,3-dihydrobenzofuran-2-yl]-2-methoxy-phenol	50.76	0.39
MOL009055	Hirsutin_qt	49.81	0.37
MOL009057	Liriodendrin_qt	53.14	0.8
MOL000098	Quercetin	46.43	0.28
MOL002773	Beta-carotene	37.18	0.58
MOL008240	(E)-3-[4-[(1R,2R)-2-Hydroxy-2-(4-hydroxy-3-methoxy-phenyl)-1-methylol-ethoxy]-3-methoxy-phenyl]acrolein	56.32	0.36
MOL011604	Syringetin	36.82	0.37

**Table 3:** Eucommiae Cortex bioactive compound-related osteoporosis targets.

GENE NAME	Protein name	ENTRY
AHR	Aryl hydrocarbon receptor	P35869
AKT1	RAC-alpha serine/threonine-protein kinase	P31749
ALB	Serum albumin	P02768
ALOX5	Arachidonate 5-lipoxygenase	P09917
AR	Androgen receptor	P10275
BAX	Apoptosis regulator BAX	Q07812
BCL2	Apoptosis regulator Bcl-2	P10415
BCL2L1	Bcl-2-like protein 1	Q07817
BIRC5	Baculoviral IAP repeat-containing protein 5	O15392
CASP3	Caspase-3	P42574
CASP8	Caspase-8	Q14790
CASP9	Caspase-9	P55211
CAV1	Caveolin-1	Q03135
CCL2	C-C motif chemokine 2	P13500
CCNB1	G2/mitotic-specific cyclin-B1	P14635
CCND1	G1/S-specific cyclin-D1	P24385
CD40LG	CD40 ligand	P29965
CDKN1A	Cyclin-dependent kinase inhibitor 1	P38936
CHRM3	Muscarinic acetylcholine receptor M3	P20309
IKKA	Inhibitor of nuclear factor kappa-B kinase subunit alpha	O15111
CLDN4	Claudin-4	O14493
CRP	C-reactive protein	P02741
CTNNB1	Catenin beta-1	P35222
CXCL2	C-X-C motif chemokine 2	P19875
CXCL8	Interleukin-8	P10145
CYP1A1	Cytochrome P450 1A1	P04798
CYP1A2	Cytochrome P450 1A2	P05177
CYP3A4	Cytochrome P450 3A4	P08684
DUOX2	Dual oxidase 2	Q9NRD8
EGF	Pro-epidermal growth factor	P01133
EGFR	Epidermal growth factor receptor	P00533
ERBB2	Receptor tyrosine-protein kinase erbB-2	P04626
ERBB3	Receptor tyrosine-protein kinase erbB-3	P21860
F3	Tissue factor	P13726
FOS	Proto-oncogene c-Fos	P01100
GJA1	Gap junction alpha-1 protein	P17302
GSK3B	Glycogen synthase kinase-3 beta	P49841
GSTM1	Glutathione S-transferase Mu 1	P09488
GSTP1	Glutathione S-transferase P	P09211
HIF1A	Hypoxia-inducible factor 1-alpha	Q16665
HSPB1	Heat shock protein beta-1	P04792
ICAM1	Intercellular adhesion molecule 1	P05362
IFNG	Interferon gamma	P01579
IGF2	Insulin-like growth factor II	P01344
IKBKB	Inhibitor of nuclear factor kappa-B kinase subunit beta	O14920
IL10	Interleukin-10	P22301
IL1A	Interleukin-1 alpha	P01583
IL1B	Interleukin-1 beta	P01584
IL2	Interleukin-2	P60568
IL6	Interleukin-6	P05231
JUN	Transcription factor AP-1	P05412
MAPK1	Mitogen-activated protein kinase 1	P28482
MAPK14	Mitogen-activated protein kinase 14	Q16539
MAPK8	Mitogen-activated protein kinase 8	P45983
MMP1	Interstitial collagenase	P03956
MMP2	72 kDa type IV collagenase	P08253
MMP3	Stromelysin-1	P08254
MMP9	Matrix metalloproteinase-9	P14780
MPO	Myeloperoxidase	P05164
MYC	Myc proto-oncogene protein	P01106
NFE2L2	Nuclear factor erythroid 2-related factor 2	Q16236
NFKBIA	NF-kappa-B inhibitor alpha	P25963
NOS2	Nitric oxide synthase, inducible	P35228
NOS3	Nitric oxide synthase, endothelial	P29474
NQO1	NAD(P)H dehydrogenase [quinone] 1	P15559
ODC1	Ornithine decarboxylase	P11926
PARP1	Poly [ADP-ribose] polymerase 1	P09874
PGR	Progesterone receptor	P06401
PLAT	Tissue-type plasminogen activator	P00750
PLAU	Urokinase-type plasminogen activator	P00749
PPARG	Peroxisome proliferator-activated receptor gamma	P37231
PTGS1	Prostaglandin G/H synthase 1	P05979
PTGS2	Prostaglandin G/H synthase 2	P35354
RASSF1	Ras association domain-containing protein 1	Q9NS23
RB1	Retinoblastoma-associated protein	P06400
SELE	E-selectin	P16581
SERPINE1	Plasminogen activator inhibitor 1	P05121
SLC6A4	Sodium-dependent serotonin transporter	P31645
SLPI	Antileukoproteinase	P03973
SOD1	Superoxide dismutase [Cu-Zn]	P00441
SPP1	Osteopontin	P10451
THBD	Thrombomodulin	P07204
TP53	Cellular tumor antigen p53	P04637
VCAM1	Vascular cell adhesion protein 1	P19320
VEGFA	Vascular endothelial growth factor A	P15692

**Table 4:** Binding energy of the selected compounds to the core genes.

	Quercetin	Kaempferol	Beta-carotene
EGF	-8.6	-5.4	-6.4
VEGFA	-10.2	-7.4	-10.2
EGFR	-9.8	-6.5	-7.3
MAPK1/ERK2	-7.9	-7.4	-8.8
C-MYC	-7.2	-8.9	-11
C-FOS	-10.4	-9.2	-8.4
CASP3	-9.6	-10.1	-9.8
AKT1	-8.1	-8.3	-8.7
MAPK8/JNK1	-6.3	-10.2	-7.7
C-JUN	-8.9	-8.5	-8.3

## Data Availability

The data used to support the findings of this study are included within the article.

## References

[1] Barnsley J., Buckland G., Chan P. E. (2021). Pathophysiology and treatment of osteoporosis: challenges for clinical practice in older people. *Aging Clinical and Experimental Research*.

[2] Sözen T., Özışık L., Başaran N. Ç. (2017). An overview and management of osteoporosis. *European Journal of Rheumatology*.

[3] Johnell O., Kanis J. A. (2006). An estimate of the worldwide prevalence and disability associated with osteoporotic fractures. *Osteoporosis international: a journal established as result of cooperation between the European Foundation for Osteoporosis and the National Osteoporosis Foundation of the USA*.

[4] Deardorff W. J., Cenzer I., Nguyen B., Lee S. J. (2022). Time to benefit of bisphosphonate therapy for the prevention of fractures among postmenopausal women with osteoporosis: a meta-analysis of randomized clinical trials. *JAMA Internal Medicine*.

[5] Khosla S., Hofbauer L. C. (2017). Osteoporosis treatment: recent developments and ongoing challenges. *The lancet Diabetes & endocrinology*.

[6] Pan Y., Niu Y., Li C. (2014). Du-zhong (Eucommia ulmoides) prevents disuse-induced osteoporosis in hind limb suspension rats. *The American Journal of Chinese Medicine*.

[7] Zhao J., Yang T., Zhao N. (2020). Research progress on osteogenic differentiation of bone marrow mesenchymal stem cells induced by eucommia ulmoides to prevent and cure osteoporosis related signal pathways. *Chinese Journal of Osteoporosis*.

[8] Wei M., Li H., Li Q. (2020). Based on network pharmacology to explore the molecular targets and mechanisms of Gegen Qinlian decoction for the treatment of ulcerative colitis. *BioMed Research International*.

[9] Wang J., Peng W., Wu F. X. (2013). Computational approaches to predicting essential proteins: a survey. *Proteomics Clinical Applications*.

[10] Qadir A., Liang S., Wu Z., Chen Z., Hu L., Qian A. (2020). Senile osteoporosis: the involvement of differentiation and senescence of bone marrow stromal cells. *International Journal of Molecular Sciences*.

[11] El-Gazzar A., Högler W. (2021). Mechanisms of bone fragility: from osteogenesis imperfecta to secondary osteoporosis. *International Journal of Molecular Sciences*.

[12] Qi S., Zheng H., Chen C., Jiang H. (2019). Du-Zhong (Eucommia ulmoides Oliv.) cortex extract alleviates lead acetate-induced bone loss in rats. *Biological Trace Element Research*.

[13] Guo Y.-J., Pan W.-W., Liu S.-B., Shen Z.-F., Xu Y., Hu L.-L. (2020). ERK/MAPK signalling pathway and tumorigenesis. *Experimental and Therapeutic Medicine*.

[14] Wen X., Jiao L., Tan H. (2022). MAPK/ERK pathway as a central regulator in vertebrate organ regeneration. *International Journal of Molecular Sciences*.

[15] Keshet Y., Seger R. (2010). The MAP kinase signaling cascades: a system of hundreds of components regulates a diverse array of physiological functions. *Methods in Molecular Biology*.

[16] Matsuguchi T., Chiba N., Bandow K., Kakimoto K., Masuda A., Ohnishi T. (2009). JNK activity is essential for Atf4 expression and late-stage osteoblast differentiation. *Journal of Bone and Mineral Research: the Official Journal of the American Society for Bone and Mineral Research*.

[17] Xu R., Zhang C., Shin D. Y. (2017). C-Jun N-terminal kinases (JNKs) are critical mediators of osteoblast activity in vivo. *Journal of Bone and Mineral Research: the Official Journal of the American Society for Bone and Mineral Research*.

[18] Hess J., Angel P., Schorpp-Kistner M. (2004). AP-1 subunits: quarrel and harmony among siblings. *Journal of Cell Science*.

[19] Wagner E. F. (2002). Functions of AP1 (Fos/Jun) in bone development. *Annals of the Rheumatic Diseases*.

[20] Zenz R., Eferl R., Scheinecker C. (2007). Activator protein 1 (Fos/Jun) functions in inflammatory bone and skin disease. *Arthritis Research & Therapy*.

[21] Wong S. K., Chin K.-Y., Ima-Nirwana S. (2020). Quercetin as an agent for protecting the bone: a review of the current evidence. *International Journal of Molecular Sciences*.

[22] Charkos T. G., Liu Y., Oumer K. S., Vuong A. M., Yang S. (2020). Effects of *β*-carotene intake on the risk of fracture: a Bayesian meta-analysis. *BMC Musculoskeletal Disorders*.

[23] Nishide Y., Tousen Y., Tadaishi M. (2015). Combined effects of soy isoflavones and *β*-carotene on osteoblast differentiation. *International Journal of Environmental Research and Public Health*.

